# Exercise as a therapeutic approach to alleviate diabetic kidney disease: mechanisms, clinical evidence and potential exercise prescriptions

**DOI:** 10.3389/fmed.2024.1471642

**Published:** 2024-10-25

**Authors:** Rao Fan, Jianda Kong, Jiahao Zhang, Lei Zhu

**Affiliations:** College of Sports Science, Qufu Normal University, Qufu, China

**Keywords:** diabetic kidney disease, exercise, blood glucose stability, RAAS, oxidative stress, inflammation, musclekidney interaction, endothelial cell function

## Abstract

Diabetic kidney disease (DKD) is a global and severe complication that imposes a significant burden on individual health, families, and society. Currently, the main treatment approaches for DKD include medication, blood glucose control, protein-restricted diet, and blood pressure management, all of which have certain limitations. Exercise, as a non-pharmacological intervention, has attracted increasing attention. This review introduces the mechanisms and clinical evidence of exercise on DKD, and proposes potential exercise prescriptions. Exercise can improve blood glucose stability related to DKD and the renin-angiotensin-aldosterone system (RAAS), reduce renal oxidative stress and inflammation, enhance the crosstalk between muscle and kidneys, and improve endothelial cell function. These mechanisms contribute to the comprehensive improvement of DKD. Compared to traditional treatment methods, exercise has several advantages, including safety, effectiveness, and no significant side effects. It can be used as an adjunct therapy to medication, blood glucose control, protein-restricted diet, and blood pressure management. Despite the evident benefits of exercise in DKD management, there is still a lack of large-scale, long-term randomized controlled trials to provide more evidence and develop exercise guidelines for DKD. Healthcare professionals should actively encourage exercise in DKD patients and develop personalized exercise plans based on individual circumstances.

## 1 Introduction

Diabetic kidney disease (DKD) is a severe complication on a global scale, posing a threat not only to individual health but also imposing a significant burden on families and societies (1, 2). Approximately 400 million people worldwide are estimated to have diabetes, and about one-third of diabetic patients eventually develop DKD, making it one of the leading causes of end-stage renal disease (ESRD) (1, 3). With the increasing number of diabetes patients, DKD has become one of the primary causes of chronic kidney disease globally.

Currently, the main treatment options for DKD include medication (4, 5), blood glucose control (4, 6), protein-restricted diet (4, 7), and blood pressure management (4, 8), lipid regulation, and antiplatelet therapy. In recent years, in addition to traditional approaches such as medication, blood glucose control, blood pressure management, and protein restriction, the roles of lipid regulation and antiplatelet therapy in the management of diabetic kidney disease (DKD) have also gained widespread recognition. Although these treatment methods have shown some effects in controlling blood glucose and blood pressure, their limitations are evident. Certain medications may lead to adverse reactions, and these methods cannot fully reverse or prevent the progression of DKD (9). Therefore, finding more effective treatment methods is of significant importance for managing and improving DKD.

In recent years, exercise has garnered increasing attention as a non-pharmacological intervention. Exercise not only improves overall metabolic control, lowers blood glucose and lipid levels, but also enhances cardiovascular health and muscle function (10). Compared to traditional treatment methods, exercise has many advantages. It is a relatively safe treatment approach with almost no apparent side effects. Exercise can also improve cardiovascular function, regulate blood pressure and circulation, thereby helping to alleviate the burden on the kidneys. Furthermore, exercise can enhance the body’s redox status, suppress inflammatory responses, and mitigate cellular oxidative stress on the kidneys (11). These advantages make exercise a potential, feasible, and effective method for treating DKD.

This review aims to delve into the role of exercise in managing DKD and comprehensively analyze the advantages and limitations of exercise compared to traditional treatment methods. By synthesizing existing clinical and experimental research, we will provide a comprehensive understanding of the mechanisms and effects of exercise in improving DKD. This will contribute to further clinical practice and guidance, providing more effective management strategies for DKD patients, improving their quality of life, and reducing the burden on families and societies.

## 2 Methods

### 2.1 Study design

This review employs a systematic literature review approach aimed at exploring the effects of exercise on diabetic kidney disease (DKD). We prioritize high-quality study designs, including randomized controlled trials (RCTs) and prospective cohort studies, to enhance the credibility of the findings.

### 2.2 Literature selection

Data were systematically retrieved from electronic databases such as PubMed, Embase, and Cochrane Library using relevant keywords, including “diabetic kidney disease,” “exercise,” and “blood glucose stability.” The study selection and data extraction were conducted by two independent researchers to ensure the accuracy of the results.

Inclusion Criteria: (i) Study Population: Participants must be diagnosed with diabetic kidney disease (DKD) according to the diagnostic criteria set by the International Diabetes Federation (IDF) or other authoritative guidelines. (ii) Study Design: We included randomized controlled trials (RCTs), prospective cohort studies, systematic reviews, and meta-analyses to ensure a comprehensive and high-quality assessment. (iii) Intervention Content: Studies must include an exercise intervention with clearly described types of exercise (e.g., aerobic, resistance training), frequency, and duration. (iv) Related Disease Studies: Research that includes other chronic diseases related to DKD (e.g., diabetes with heart disease, hypertension) to provide broader context. (v) Outcome Assessment: Studies should report specific effects of exercise on DKD patients, including blood glucose stability, renal function indicators (such as creatinine and urine protein), and related pathological mechanisms.

Exclusion Criteria: (i) Comorbid Patients: Studies focusing on patients with severe unrelated diseases (e.g., end-stage renal disease, active cancer) will be excluded to reduce confounding factors. (ii) Healthy Individuals: Exclude studies that involve healthy individuals as participants without mentioning DKD patients. (iii) Insufficient Sample Size: Exclude studies with fewer than 100 participants to ensure the validity of statistical analyses. (iv) Lack of Long-term Follow-up: Exclude studies that do not provide at least 6 months of follow-up data to assess the long-term effects of exercise.

### 2.3 Sample size

We prefer studies with larger sample sizes to ensure statistical significance and representativeness. The selected literature included studies with sample sizes exceeding 100 participants, providing sufficient statistical support.

### 2.4 Data collection

Quantitative analyses were conducted on the results of the included studies, employing appropriate statistical methods to evaluate the impact of exercise on DKD patients and further explore the effects of different exercise interventions on the underlying pathological mechanisms.

## 3 Results

### 3.1 Pathogenesis of DKD

**Figure 1:**
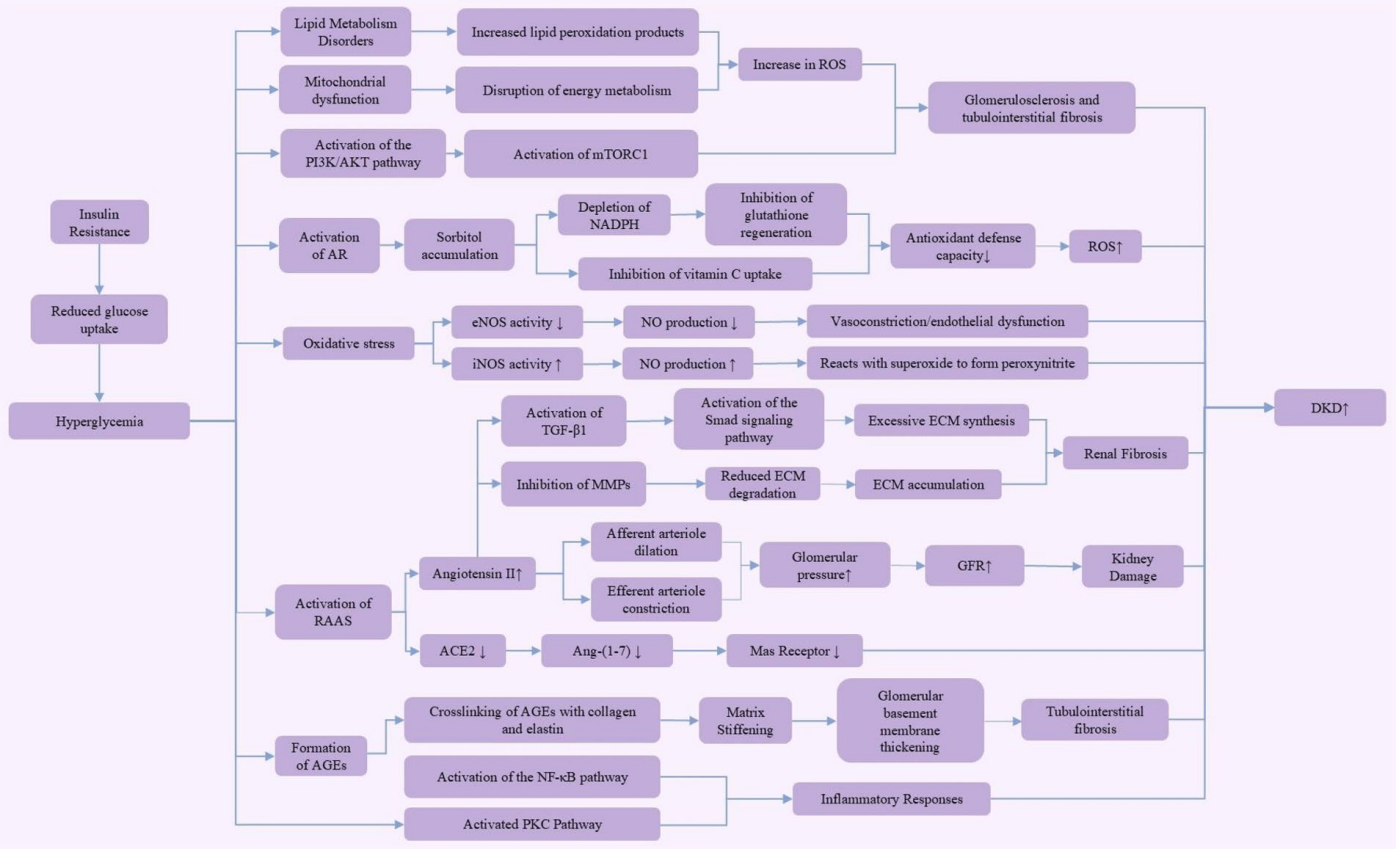
Pathogenesis of diabetic kidney disease (DKD). This figure illustrates the multiple pathogenic mechanisms of hyperglycemia in the development of diabetic kidney disease (DKD). Hyperglycemia, through insulin resistance, leads to reduced glucose uptake, activating various pathways, including the aldose reductase (AR) pathway, PI3K/AKT pathway, and mammalian target of rapamycin complex 1 (mTORC1) pathway. These pathways contribute to lipid metabolism disorders, mitochondrial dysfunction, and increased reactive oxygen species (ROS) production. These metabolic changes further result in diminished antioxidant defense capacity and enhanced extracellular matrix (ECM) synthesis, ultimately leading to glomerulosclerosis, renal fibrosis, and tubulointerstitial fibrosis. Activation of the renin-angiotensin-aldosterone system (RAAS) increases glomerular pressure and glomerular filtration rate (GFR), exacerbating kidney damage. Additionally, the accumulation of advanced glycation end products (AGEs) through crosslinking with collagen and elastin activates the nuclear factor-kappa B (NF-κB) and protein kinase C (PKC) pathways, driving inflammatory responses. These complex mechanisms collectively promote the onset and progression of diabetic kidney disease.

Diabetic kidney disease (DKD) is a chronic complication of diabetes and the leading cause of end-stage renal disease. Its pathogenesis is complex, involving various molecular and cellular pathways, with hyperglycemia and hemodynamic disturbances being the primary driving factors for its onset and progression (Figure 1).

In DKD, dysfunction of the afferent and efferent arterioles is a key factor leading to increased glomerular filtration rate (GFR). Under normal conditions, the afferent arteriole, located at the entrance of the glomerulus, delivers blood to the glomerulus. Its dilation increases blood flow and pressure within the glomerulus, thereby enhancing the filtration rate. The efferent arteriole, situated at the exit, carries filtered blood away from the glomerulus; its constriction further increases glomerular pressure, leading to a rise in GFR (12). Metabolic disturbances in DKD, along with oxidative stress, inhibit nitric oxide (NO) production, which typically reduces vasodilation. However, in a hyperglycemic environment, the activation of the local renin-angiotensin system (RAS) elevates angiotensin II levels, causing efferent arteriole constriction. As a compensatory mechanism, afferent arteriole dilation occurs to maintain glomerular filtration. This combination of afferent dilation and efferent constriction significantly increases intraglomerular pressure, thereby raising GFR, exacerbating kidney damage, and leading to proteinuria (13–15).

In the early stages of DKD, persistent hyperglycemia induces changes in vascular factors, such as NO and endothelin-1, leading to the aforementioned vascular dysfunction. As intraglomerular pressure continues to rise, hyperfiltration develops, laying the foundation for subsequent kidney damage. These hemodynamic changes also cause phenotypic alterations in endothelial cells, increasing the permeability of the glomerular basement membrane, eventually resulting in proteinuria (13). In addition to hemodynamic changes, hyperglycemia exacerbates oxidative stress through the activation of aldose reductase (AR) in the polyol pathway. This pathway converts excess glucose into sorbitol, which accumulates within cells, consuming NADPH and inhibiting glutathione (GSH) regeneration. Since NADPH is an essential cofactor for GSH reduction, its depletion weakens the cells ability to scavenge reactive oxygen species (ROS), thereby intensifying oxidative stress (16). Moreover, sorbitol and vitamin C share the same cellular transport channel, so elevated sorbitol levels can inhibit vitamin C uptake, impairing the cell’s antioxidant defense capacity (17). Studies have shown that increased AR activity is closely linked to enhanced ROS production and the progression of DKD (18). Thus, AR not only compromises antioxidant defenses by depleting NADPH but also directly increases ROS production through sorbitol metabolism (17, 19). Consequently, the use of aldose reductase inhibitors has been considered a potential therapeutic strategy for DKD intervention, as they effectively reduce ROS levels (19, 20). As DKD progresses, alterations in intracellular glucose metabolism lead to the formation of advanced glycation end products (AGEs), further driving DKD progression. In a hyperglycemic environment, non-enzymatic glycation produces AGEs that cross-link with collagen and elastin in the extracellular matrix, altering their molecular structure and elasticity, thereby increasing matrix stiffness (21). This stiffening is a major cause of glomerular basement membrane thickening and tubulointerstitial fibrosis, disrupting normal kidney architecture and contributing to chronic kidney disease (CKD) (22). These structural changes play a key role in the pathophysiology of diabetes-related complications. Matrix stiffening limits the normal expansion and contraction of renal tubules, leading to impaired filtration and reabsorption functions, thereby accelerating DKD progression (23, 24). Furthermore, the cross-linking of AGEs with collagen and elastin not only affects the mechanical properties of renal tubules but also activates fibrotic pathways in the kidneys through oxidative stress and inflammatory responses (25). This fibrosis is a key factor in the pathological progression of DKD, further impairing normal kidney structure and function (25, 26). Research indicates that transforming growth factor-β1 (TGF-β1) is a key mediator of renal fibrosis in DKD. TGF-β1 primarily activates the Smad signaling pathway, promoting the transdifferentiation of renal intrinsic cells, including tubular epithelial cells, mesangial cells, and fibroblasts, into myofibroblasts. These myofibroblasts are the main source of excessive extracellular matrix (ECM) synthesis, producing and secreting ECM components like type I and IV collagen, fibronectin, and laminin (27, 28). Additionally, TGF-β1 inhibits the activity of matrix metalloproteinases (MMPs), reducing ECM degradation and resulting in ECM accumulation in kidney tissue (29, 30). This excessive ECM deposition ultimately leads to structural disorganization of the kidney, forming scar tissue that restricts normal kidney function (28, 29). Therefore, TGF-β1 serves as a crucial driver of fibrosis in DKD. Aside from hyperglycemia-induced pathological changes, lipid metabolism disorders can further worsen the condition by promoting ROS production through increased lipid peroxidation products. This process leads to glomerulosclerosis and tubulointerstitial fibrosis, aggravating DKD severity (31, 32). Moreover, AGEs enhance the expression of various NF-κB-related pro-inflammatory mediators, activated by ROS produced in high-glucose environments. Podocyte exposure to AGEs results in the upregulation of pro-inflammatory cytokines, infiltration of inflammatory cells, and stimulation of adhesion molecules and profibrotic factors (33). With the accumulation of AGEs and persistent hyperglycemia, the renin-angiotensin-aldosterone system (RAAS) is activated. RAAS activation results in the production of angiotensin II (Ang II), which primarily induces oxidative stress via NADPH oxidase and promotes inflammation and fibrosis through pro-inflammatory and profibrotic cytokines (34, 35). The pathogenesis of DKD is complex, involving interactions at multiple biological levels. At the molecular level, genetic and epigenetic regulation plays a significant role in DKD progression. Studies indicate that changes in gene expression and epigenetic modifications, such as DNA methylation, histone changes, and non-coding RNA activity, are closely associated with inflammation and fibrosis-related signaling pathways (36, 37). These mechanisms are influenced by hyperglycemia-induced oxidative stress and AGE formation, accelerating the pathological process of DKD. At the cellular level, mitochondrial dysfunction, along with disturbances in energy metabolism and excessive ROS production, are key contributors to DKD (36, 38). Furthermore, dysregulation of the autophagy process worsens cellular damage and compromises the integrity of the glomerular filtration barrier, leading to proteinuria (36, 38). At the tissue level, renal fibrosis is a hallmark of advanced DKD, where ongoing inflammation and ECM accumulation lead to tubulointerstitial fibrosis, causing irreversible damage to kidney structure and function (36). These insights provide a more comprehensive understanding of the mechanisms underlying DKD.

### 3.2 Mechanistic role of exercise in improving DKD

**Figure 2:**
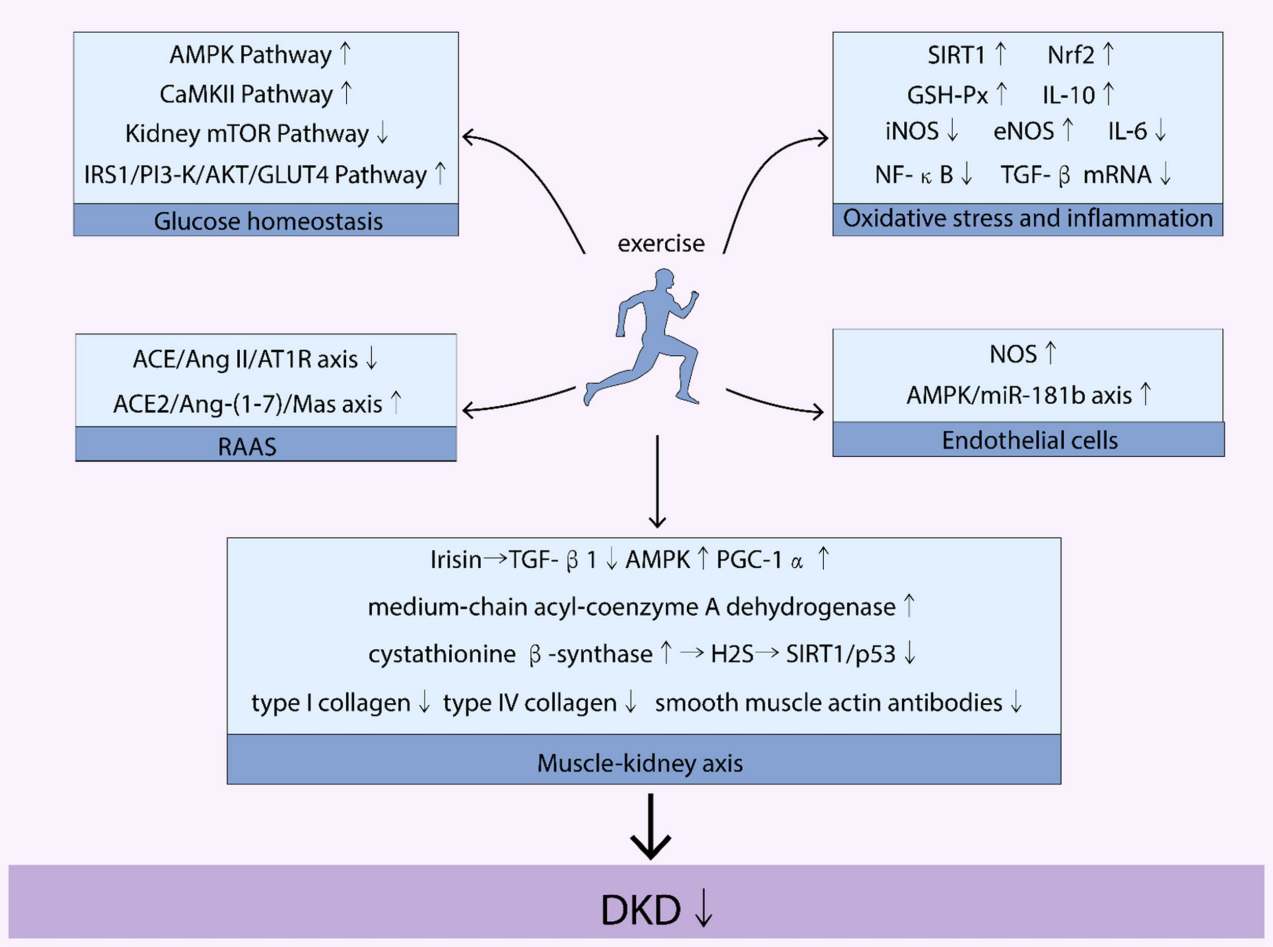
Mechanisms of the benefits of exercise on DKD. Figure 1 illustrates how exercise affects various pathways and axes related to diabetic kidney disease (DKD). Key pathways and molecules involved include the AMP-activated protein kinase (AMPK) pathway, mammalian target of rapamycin (mTOR) pathway, calcium/calmodulin-dependent protein kinase II (CaMKII) pathway, and the insulin receptor substrate 1/phosphoinositide 3-kinase/protein kinase B/glucose transporter type 4 (IRS1/PI3-K/AKT/GLUT4) pathway. It also highlights the role of sirtuin 1 (SIRT1), nuclear factor erythroid 2-related factor 2 (Nrf2), glutathione peroxidase (GSH-Px), interleukins IL-6 and IL-10, inducible nitric oxide synthase (iNOS), endothelial nitric oxide synthase (eNOS), nuclear factor kappa-light-chain-enhancer of activated B cells (NF-κB), and transforming growth factor beta (TGF-β). The renin-angiotensin-aldosterone system (RAAS) is also depicted, with specific focus on the angiotensin-converting enzyme 2/angiotensin II/angiotensin II receptor type 1 (ACE/Ang II/AT1R) axis and the angiotensin-converting enzyme 2/angiotensin-(1-7)/Mas receptor (ACE2/Ang-(1-7)/Mas) axis. Additionally, the nitric oxide synthase (NOS) and AMP-activated protein kinase/microRNA-181b (AMPK/miR-181b) axis are mentioned. The image further addresses the roles of transforming growth factor beta 1 (TGF-β1), peroxisome proliferator-activated receptor gamma coactivator 1-alpha (PGC-1α), and diabetic kidney disease (DKD).

Exercise serves as a crucial intervention in the management of diabetic kidney disease (DKD), particularly in stabilizing blood glucose levels, a key factor in the progression of DKD in patients with Type 2 diabetes mellitus (T2DM). The mechanistic role of exercise in improving DKD, as illustrated in Figure 2, is multifaceted, involving various pathways that collectively contribute to better blood glucose stability. These pathways include the CaMKII, AMPK, mTOR, and IRS1/PI3-K/AKT/GLUT4 pathways, each playing a unique role in glucose metabolism and insulin sensitivity.

By engaging in regular physical activity, patients with DKD can activate these pathways, leading to improved insulin sensitivity, enhanced glucose uptake by skeletal muscles, and reduced blood glucose levels. For instance, the AMPK pathway, a well-known energy sensor, is particularly responsive to exercise. Activation of this pathway through physical activity improves insulin sensitivity and glucose uptake, crucial for maintaining stable blood glucose levels. Similarly, exercise-induced activation of the IRS1/PI3-K/AKT/GLUT4 pathway enhances the efficiency of glucose transport into cells, further contributing to blood glucose stability.

Moreover, exercise influences the regulation of blood glucose homeostasis by affecting muscle contraction and energy utilization. For example, muscle contractions during exercise stimulate the translocation of glucose transporters to the cell membrane, facilitating glucose entry into cells. This process is crucial for maintaining blood glucose levels within a normal range, especially in individuals with T2DM, who are at a higher risk of developing DKD.

In summary, the role of exercise in improving DKD extends beyond physical fitness. It involves a complex interplay of metabolic pathways that work together to stabilize blood glucose levels. This stabilization is key in managing DKD, as it directly impacts the progression of kidney disease in patients with T2DM. The beneficial effects of exercise on these pathways underscore its importance as a therapeutic strategy in the treatment and management of DKD. This section provides a comprehensive review of the mechanistic role of exercise in improving DKD.

#### 3.2.1 Exercise improves blood glucose stability associated with DKD

##### 3.2.1.1 Occurrence of DKD in patients with T2DM is associated with blood glucose homeostasis

Type 2 diabetes mellitus (T2DM) is a progressive disease characterized by reduced insulin secretion, increased insulin resistance, and disorders of glucagon metabolism. The persistent high-glucose environment in the body can lead to diabetic kidney disease (DKD) (39). Therefore, T2DM serves as a precursor to the occurrence of DKD. Early research that insulin receptor substrate (IRS), as a key mediator of insulin signaling transduction, can promote glucose uptake by skeletal muscle (40). When IRS1 is knocked out, mice exhibit peripheral insulin resistance and growth retardation. When IRS2 is knocked out, metabolic defects occur in the liver, skeletal muscle, and fat, accompanied by apoptosis of islet β-cells (41). Moreover, activated AMP-activated protein kinase (AMPK) in skeletal muscle promotes the transfer glucose transporter 4 (GLUT4) from the intracellular pool to the cell membrane, thereby facilitating glucose entry into cells (42). However, Jiang et al. pointed out that under high-glucose conditions, AMPK and liver kinase B1 (LKB1) undergo dissociation (43). Furthermore, studies have also found that in the skeletal muscle of obese mice fed a high-fat diet, activated mammalian target of rapamycin (mTOR) inhibits downstream insulin signaling mediated by S6K1, leading to degradation of IRS1 and IRS2, reduced glucose uptake in skeletal muscle, and glycogen accumulation (44). Additionally, Williamson et al. reported that the phosphorylation level of S6K1 in skeletal muscle of T2DM patients is higher (45).

In conclusion, IRS1 and IRS2 are crucial for maintaining blood glucose homeostasis. However, under high-glucose conditions, AMPK levels decrease and its signaling pathway is inhibited, while mTOR levels increase and its signaling pathway is activated.

##### 3.2.1.2 Pathways involved in the regulation of blood sugar homeostasis by exercise

###### 3.2.1.2.1 CaMKII pathway

Muscle contraction can increase intracellular Ca^2+^ concentration and the amount of calcium regulatory protein complexes in the body. In the Ca^2+^-related calcium regulatory protein signaling pathway, calcium/calmodulin-dependent protein kinase II (CaMKII) is also an important molecule for skeletal muscle glucose uptake (46). When inhibiting CaMKII gene expression, although the activity of CaMK decreased by 35% and glucose uptake decreased by 30%, the level of GLUT4 protein did not change, while AMPK phosphorylation significantly increased, which is derived from the study of Witczak et al. (47). In addition, Smith et al. (48) demonstrated that aerobic exercise can increase the phosphorylation of CaMKII around rat skeletal muscle fibers and increase GLUT4 mRNA levels by 2.2-fold and protein levels by 1.8-fold. More encouragingly, Combes et al. (49) confirmed that compared to resting levels, healthy subjects undergoing high-intensity interval training (HIIT) had increased phosphorylation levels of AMPK and CaMKII in calf muscle by 2.9-fold and 2.7-fold, respectively. However, after 3 h of retesting, there was no further increase in the phosphorylation levels of AMPK and CaMKII. Therefore, it can be concluded that a certain intensity of exercise is required to better activate CaMKII, and there may be some overlap between CaMKII and AMPK in regulating glucose metabolism in the body.

###### 3.2.1.2.2 AMPK pathway

Studies have demonstrated that exercise can enhance glucose homeostasis in patients with diabetic kidney disease (DKD) by activating the AMP-activated protein kinase (AMPK) pathway. AMPK serves as a critical energy-sensing enzyme that regulates cellular energy balance and metabolism (50). The AMPK pathway plays a key role in managing both diabetes and kidney diseases. In the context of DKD, AMPK activation can improve insulin sensitivity and promote glucose uptake and utilization, contributing to more stable blood glucose levels (51). For instance, regular aerobic exercise significantly activates the AMPK pathway, leading to improved blood glucose control and enhanced renal function in patients with type 2 diabetes (52). Exercise-induced activation of AMPK reduces inflammation and oxidative stress through multiple mechanisms. First, AMPK inhibits the activation of nuclear factor-κB (NF-κB) by suppressing IκB kinase (IKK), which decreases the production of pro-inflammatory cytokines, such as TNF-α, IL-6, and IL-1β. This process effectively reduces systemic inflammatory responses (53, 54). Second, AMPK activation upregulates nuclear factor erythroid 2–related factor 2 (Nrf2), a key antioxidant transcription factor. Nrf2 enhances the expression of antioxidant enzymes like superoxide dismutase (SOD) and catalase (CAT), thereby boosting the cell’s capacity to neutralize reactive oxygen species (ROS) and mitigating oxidative stress-induced damage (55, 56). Additionally, AMPK upregulates peroxisome proliferator-activated receptor gamma coactivator 1-alpha (PGC-1α), which promotes mitochondrial biogenesis and improves mitochondrial function and efficiency. This action further reduces ROS production, thereby not only decreasing oxidative stress-related damage to organs such as the kidneys but also improving cellular metabolic homeostasis, offering protective effects against oxidative injury (53, 57). Moreover, the reduction of inflammation and oxidative stress through AMPK pathway activation is vital for slowing DKD progression. Aerobic exercise has been shown to enhance AMPK activity, thereby reducing inflammation and fibrosis in diabetic mouse models (58). Thus, exercise, through AMPK pathway activation, presents significant potential in improving glucose homeostasis and renal health in patients with DKD.

###### 3.2.1.2.3 mTOR pathway

The mTOR pathway plays a crucial role in regulating cell growth and metabolism by integrating nutritional signals, energy status, and growth factor signals to modulate protein synthesis and cellular growth. In diabetic kidney disease (DKD), excessive activation of mTORC1 is closely related to the occurrence of glomerulosclerosis and fibrosis, which further leads to renal dysfunction, including damage to podocytes and proximal tubular cells (59, 60). Recent studies have shown that mTOR inhibitors can block the mTOR pathway while simultaneously triggering anti-inflammatory, anti-proliferative, and autophagy-inducing responses in the body. Generally, mTOR is activated in various disease states (61). Research evidence also indicates that exercise training can reduce insulin resistance in the myocardium of diet-induced obese rats and upregulate the mTOR/p70S6k pathway (62). It is noteworthy that different types of exercise exert distinct effects on the mTOR pathway. In normal rats, both aerobic and resistance exercises can increase mTOR phosphorylation levels; however, resistance exercise has been shown to induce a greater degree of phosphorylation compared to aerobic exercise (63). An earlier study observed that, after exercise, the phosphorylation levels of mTOR in both the exercised and non-exercised legs of healthy participants increased by 45–65%, alongside a 40% increase in AMPK phosphorylation levels (64). In disease states, the mTOR pathway is typically abnormally activated, contributing to inflammation and metabolic dysfunction. However, during exercise, mTOR activation plays a critical role in normal cellular repair and protein synthesis, facilitating muscle and tissue recovery and regeneration (65, 66). The tissue-specific effects of exercise are particularly significant in DKD patients. Exercise activates the AMPK pathway, which subsequently inhibits excessive mTORC1 activity, thereby reducing oxidative stress and fibrosis in the kidneys and improving renal function (60, 67). In contrast, in skeletal muscle, exercise has a different impact. Resistance training and high-intensity interval training (HIIT) can activate mTOR, promoting protein synthesis and supporting muscle growth and repair (68). This activation is driven by mechanical tension and growth factor stimulation, leading to muscle fiber hypertrophy and increased strength. Post-exercise, the enhanced mTOR activity also facilitates the absorption and utilization of nutrients by muscle cells, strengthening muscle metabolism (68, 69). Therefore, in healthy individuals, the increased phosphorylation of mTOR induced by exercise may be necessary for certain metabolic processes. Although our understanding of the regulatory relationship between exercise and the mTOR pathway has advanced, direct evidence on how the mTOR pathway is regulated in DKD patients or animal models remains limited, warranting further investigation to elucidate the underlying mechanisms.

###### 3.2.1.2.4 IRS1/PI3-K/AKT/GLUT4 pathway

According to research by Kirwan et al. (70), 10 weeks of aerobic exercise can increase the expression of IRS1 protein in insulin-resistant rats. Additionally, it has been suggested that regular aerobic exercise can increase the activity level of PI3K, which is related to IRS1, in human skeletal muscle, and promote the uptake of glucose by GLUT4 in skeletal muscle (71). Furthermore, evidence has shown that moderate aerobic exercise not only increases the content and phosphorylation level of IRS2, but also promotes the sustained growth effect of GLUT4 protein content and IRS2 phosphorylation (72). Additionally, studies have found that aerobic exercise can promote the expression of IRS1, PI3K, AKT, GLUT4, etc. (73). These proteins form the IRS1/PI3K/AKT/GLUT4 pathway, thereby enhancing insulin sensitivity (74, 75). Therefore, exercise can improve peripheral insulin resistance through the IRS1/PI3K/AKT/GLUT4 pathway, which may be effective in ameliorating the progression of DKD.

#### 3.2.2 Exercise improves RAAS associated with DKD

##### 3.2.2.1 Role of RAAS activation and aldosterone in diabetic kidney disease

The renin-angiotensin-aldosterone system (RAAS) is widely distributed throughout the body and plays a critical role in maintaining blood flow stability by regulating water, electrolyte balance, and blood pressure. RAAS consists of two axes: the pressor axis and the depressor axis. In the pressor axis, renin converts angiotensinogen into angiotensin I (Ang I), which is then transformed into angiotensin II (Ang II) by angiotensin-converting enzyme (ACE). Ang II, the primary effector of the pressor axis, binds to Ang II type 1 receptor (AT1R), leading to vasoconstriction, promotion of renal water and sodium reabsorption, and aldosterone release (76, 77). In diabetic kidney disease (DKD), the RAAS-mediated activation of the pressor axis becomes sustained and excessive, resulting in increased intraglomerular pressure and glomerular hypertension. This enhanced activity of Ang II also leads to an increase in reactive oxygen species (ROS) production and extracellular matrix (ECM) accumulation, further stimulating mesangial cells to synthesize more transforming growth factor-β1 (TGF-β1). These changes ultimately contribute to glomerulosclerosis and fibrosis (78).

Studies in diabetic rats have shown that transfection with the ACE2 gene can lower blood pressure, urinary protein excretion, and kidney stiffness. This was accompanied by decreased expression of TGF-β1 and vascular endothelial growth factor (VEGF) mRNA, as well as increased superoxide dismutase (SOD) activity, Ang-(1–7) concentration, and nephrin content in the kidneys (79). Therefore, in the context of DKD, the RAAS pressor axis is excessively activated and the levels of AngII and its downstream components in the kidney are elevated, while the depressor axis and ACE2 in the kidney are inhibited. Therefore, in the context of DKD, the pressor axis of RAAS is excessively activated, elevating Ang II levels and its downstream effects in the kidney, while the depressor axis, particularly ACE2, is inhibited.

In hyperglycemic conditions, the balance between the RAAS pressor axis (ACE/Ang II/AT1R axis) and the depressor axis (ACE2/Ang-(1–7)/Mas receptor axis) is disrupted, resulting in overactivation of the pressor axis and suppression of the depressor axis (80). Hyperglycemia-induced glomerular hypertension directly stimulates the pressor axis, leading to increased Ang II levels (81). Simultaneously, oxidative stress and inflammation triggered by hyperglycemia suppress ACE2 expression, hindering the conversion of Ang II to Ang-(1–7) and further exacerbating RAAS imbalance (82, 83).

Apart from its direct effects on smooth muscle contraction to increase blood pressure, RAAS can indirectly elevate blood pressure by stimulating aldosterone production. Ang II acts on the adrenal glands to promote aldosterone synthesis (84). In diabetic rats, the mRNA expression of aldosterone synthase is reported to be 12 times higher than in control rats. However, using an AT1R antagonist significantly reduces its expression in the kidney (85). Additionally, diabetic rats that underwent bilateral adrenalectomy showed elevated glucose levels and kidney aldosterone content but had lower plasma aldosterone levels. When treated with an aldosterone synthase inhibitor, these diabetic rats exhibited reduced kidney aldosterone content, along with decreased levels of nuclear factor-κB (NF-κB) and TGF-β1 proteins, although there was no change in blood glucose levels. This indicates that the systemic aldosterone system can influence the regulation of the local renal aldosterone system (86).

Overall, in DKD, the systemic aldosterone system contributes to the regulation of the local aldosterone system in the kidneys. Increased local aldosterone exacerbates renal inflammation, further promoting the progression of DKD. However, in diabetic rats with ACE2 gene transfection, these negative effects were mitigated. Further research is needed to explore the clinical implications of modulating RAAS in DKD.

##### 3.2.2.2 Modulation of the RAAS by exercise

The influence of exercise on the RAAS has attracted widespread attention in the field of DKD. Research has shown that exercise can activate the ACE2/Ang-(1–7)/Mas axis and inhibit the activity of the ACE/AngII/AT1R axis (87). Furthermore, studies have revealed that post-exercise, the urine concentrations of AngII decrease in healthy individuals, while those of Ang-(1–7) increase (88). In studies investigating the effects of aerobic exercise and aerobic exercise combined with metformin treatment in diabetic mice, it was found that the expression level of ACE2 in the urine of the mice decreased during the second week and continued to decrease throughout the tenth week. Compared to the control group, exercise increased the expression level of ACE2 in the glomeruli of diabetic mice (89).

Another four-year follow-up survey showed that patients with diabetes who had reduced daily activity had increased glomerular filtration rate, blood creatinine levels, and glycated hemoglobin levels. However, patients with diabetes who engaged in moderate daily activities had reduced metabolic levels, thereby mitigating the adverse effects on the kidneys (90). Animal research data indicate that physical exercise can down-regulate the classic ACE/Ang II/AT1R axis and up-regulate the ACE 2/Ang1-7/Mas axis (91). Additionally, resistance training shifted the balance of the RAAS in diabetic rat kidneys toward the ACE2/Ang 1–7 axis and reduced inflammation (92). However, these findings require further validation in clinical practice.

It is noteworthy that studies on renal failure animal models have revealed that regular exercise can reduce the accumulation of AngII in the heart, alleviate left ventricular remodeling, and decrease the degree of myocardial fibrosis. This may provide assistance in improving left ventricular hypertrophy issues in patients with DKD renal failure (93).

In conclusion, exercise plays a positive role in regulating the RAAS, providing new insights into the treatment of DKD. However, further research is needed on the specific application of exercise in clinical practice.

#### 3.2.3 Exercise improves renal oxidative stress and inflammation associated with DKD

##### 3.2.3.1 DKD and renal oxidative stress and inflammation

It is interesting that mitochondria play key roles in both energy production and the generation of reactive oxygen species (ROS), as well as processes such as mitochondrial self-replication and mitochondrial differentiation. In other cells, mitochondria also regulate cell proliferation, differentiation, and inflammation (94). Research has found that respiratory reserve capacity is significantly reduced in mouse glomerular endothelial cells treated with high glucose. Diabetic mice show increased mitochondrial ROS and damaged podocyte mitosis compared to normal mice (95). Additionally, in podocytes treated with high glucose, the mRNA levels of silent information regulator 1 (Sirt1), which is associated with silent information regulator 2 (Sir2), are decreased (96). Moreover, in diabetic mice with knockdown of progranulin (PGRN) gene, the expression levels of Sirt1 and acetylation levels of PGC-1α are significantly increased.

In addition, in the case of diabetic kidney disease (DKD), protein misfolding in the endoplasmic reticulum (ER) can affect protein expression and glycosylation processes, leading to increased proteinuria and renal inflammation. Furthermore, the ER can generate a certain amount of ROS through the uncoupling reaction of nitric oxide synthase (NOS) (97). In DKD, dysfunction of glomerular endothelial cells (GECs) is an early manifestation. This is followed by sustained hyperglycemia and increased ROS in the kidney, which can inhibit NOS gene expression and reduce nitric oxide (NO) levels (98). At the same time, advanced glycation end products (AGEs) continue to accumulate in the kidneys and bind with collagen, leading to thickening of the glomerular basement membrane and induction of mesangial cell synthesis of more extracellular matrix (ECM). This can also activate nuclear factor κ-B (NF-κB), the PI3K/AKT/mTOR pathway, as well as decrease the levels of antioxidant enzymes, glutathione, and NOS (99). Moreover, some glucose can generate more fructose and reduced coenzyme I (NADH) through the polyol pathway. The increased NADH can generate more ROS through the mitochondrial electron respiratory chain, as well as generate more glycerides through glycolysis and the tricarboxylic acid cycle (100).

Sustained hyperglycemia and glycerides can activate the protein kinase C (PKC) pathway. The activated PKC pathway not only inhibits NOS gene expression but also activates the NF-κB pathway (101). Meanwhile, studies have observed increased mRNA levels of PKC, transforming growth factor-β1 (TGF-β1), and collagen proteins such as fibronectin I, fibronectin III, and collagen type IV in the renal tubulointerstitium of diabetic mice (102).

In conclusion, oxidative stress and inflammation in DKD are the results of multiple factors. Mitochondrial dysfunction, endothelial cell dysfunction, AGEs accumulation, activation of the polyol pathway, and activation of the PKC pathway are major causes of renal oxidative stress and inflammation. Under the interaction and influence of these factors, loss of renal structure and function, as well as apoptosis of related cells, can occur. Based on this, DKD can progress toward a worse outcome.

##### 3.2.3.2 Exercise reduces renal oxidative stress and inflammation

Current evidence from both direct and indirect animal studies indicates that exercise can mitigate renal oxidative stress and inflammation. For example, in an 8-week aerobic exercise program, not only was lipid peroxidation in the renal cortex of obese rats with diabetes mellitus (DM) reduced, but levels of nitric oxide (NO) and inducible nitric oxide synthase (iNOS) increased, while endothelial nitric oxide synthase (eNOS) levels decreased (103). Studies have shown that hyperglycemia-induced oxidative stress exacerbates renal injury in diabetic nephropathy by affecting the function of nitric oxide synthases, specifically eNOS and iNOS. In a hyperglycemic environment, the production of reactive oxygen species (ROS) is significantly increased, impairing eNOS activity and reducing NO bioavailability, which in turn damages vascular endothelial function (104, 105). At the same time, high glucose levels trigger an inflammatory response that promotes the overexpression of iNOS (106). The excess NO produced by iNOS combines with superoxide radicals to form peroxynitrite, a potent oxidant that further aggravates renal cell damage and accelerates the progression of DKD (105, 107). These findings suggest that aerobic exercise can alleviate renal oxidative stress and inflammation by modulating the nitric oxide system. Similarly, moderate-intensity aerobic exercise has been shown to reduce the number of macrophages and lymphocytes in the glomeruli of DM mice, while also decreasing the expression levels of NF-κB gene and TGF-β1 mRNA in renal interstitial tissue (108). Additionally, research has found that aerobic exercise upregulates the expression of SIRT1 and inhibits the acetylation of NF-κB in the kidneys of DM mice (109). This suggests that aerobic exercise may have a protective effect on renal oxidative stress and inflammation by regulating the activity of SIRT1 and NF-κB.

In clinical applications, there are also studies demonstrating the positive effects of exercise in alleviating renal oxidative stress and inflammation. There is already evidence indicating that patients with CKD have significant organ damage (110, 111). However, there have been studies attempting a 3-month resistance exercise program in patients with chronic kidney disease (CKD), which reported an increase in the expression levels of Nrf2 mRNA and glutathione peroxidase (GSH-Px) mRNA, but no significant change in the expression level of NF-κB (112). This suggests that resistance exercise may have some improvement effect on oxidative stress in CKD patients, but its impact on inflammation may be limited. In addition, a 30-min walking exercise induced a significant increase in plasma anti-inflammatory cytokine IL-10 and pro-inflammatory cytokine IL-6 levels, while maintaining a regular walking exercise for 6 months resulted in a decrease in the IL-6/IL-10 ratio in the plasma and a reduction in the activation of T lymphocytes and monocytes (113). This suggests that long-term walking exercise can produce anti-inflammatory effects and reduce the activation of immune cells, which may help alleviate renal inflammation.

Overall, animal and clinical studies demonstrate the positive effects of exercise in alleviating renal oxidative stress and inflammation. However, the differences in exercise type and intensity may have different impacts on the kidneys, and further research is needed to investigate the mechanisms and clinical application value in depth.

#### 3.2.4 Exercise improves DKD through crosstalk between muscle and kidney

The interaction between the kidney and muscle forms a muscle-kidney axis, which can be modulated by exercise. The muscle, aside from its role in movement, also functions as an endocrine organ by secreting myokines into the bloodstream, thereby exerting various biological effects (2, 13). Research has shown that muscle contraction during acute exercise releases a large amount of IL-6. However, regular and sustained exercise can significantly lower baseline levels of IL-6, reduce systemic inflammation, and delay the progression of diabetic kidney disease (DKD) (2, 114). One of the myokines, irisin, has been found to inhibit TGF-β1, thereby reducing renal fibrosis. Aerobic exercise induces the secretion of irisin, which can activate AMPK and dose-dependently inhibit high glucose-induced extracellular matrix accumulation in renal tubular epithelial cells, providing a protective effect on the kidney (114). In addition, several other myokines, including myostatin, YKL-40, fatty acid-binding proteins, and fibroblast growth factor 21, have been identified. Further research is needed to explore their potential associations with DKD (13).

Lactate, another muscle-derived factor, is produced not only during high-intensity exercise but also accumulates in muscle tissue even during low to moderate intensity exercise, potentially influencing renal function (115, 116). The kidneys play a key role in lactate clearance and metabolism, but elevated lactate levels, especially in patients with DKD, can increase the metabolic burden on the kidneys (117). Additionally, lactate may impact renal function by affecting endothelial cell function and enhancing oxidative stress (115). However, the specific effects of lactate on kidney function during moderate-intensity exercise remain to be fully understood and warrant further investigation (115, 116). Exercise may improve DKD by regulating pathways such as fatty acid metabolism, podocyte apoptosis, and renal fibrosis. Although clinical evidence supporting the effects of exercise on lipid metabolism in DKD is limited, animal studies have shown that exercise can increase the expression of medium-chain acyl-coenzyme A dehydrogenase and PGC-1α, both of which are involved in fatty acid metabolism. This can independently reduce glomerular and tubulointerstitial damage, regardless of blood glucose regulation (118). Furthermore, skeletal muscle contraction increases the body’s energy demand, promotes peripheral fat breakdown, and enhances hepatic free fatty acid oxidation, thereby improving glucose homeostasis and contributing to DKD improvement (119). Treadmill training has also been found to reverse the downregulation of cystathionine β-synthase and cystathionine γ-lyase, thereby enhancing endogenous hydrogen sulfide production in the kidney, inhibiting the SIRT1/p53 apoptosis pathway, and alleviating diabetes-associated renal injury in streptozotocin-induced diabetic mice (120). Additionally, aerobic exercise can reduce the expression of TGF-β, type I collagen, type IV collagen, and smooth muscle actin antibodies, thus slowing the progression of renal fibrosis (121).

In conclusion, muscle plays a significant role in DKD, and exercise can regulate the muscle-kidney interaction through various pathways, thereby improving DKD progression. A deeper understanding of this crosstalk and the impact of exercise on DKD can provide valuable insights into the prevention and treatment of the disease.

#### 3.2.5 Exercise improves endothelial cell function associated with DKD

Exercise improves endothelial cell function associated with DKD. Research has shown that exercise can increase the expression of nitric oxide synthase (NOS), reduce oxidative stress, and improve vascular relaxation in diabetic mice. Specifically, exercise significantly improves the expression of endothelial NOS in obese diabetic rats (122). Eight weeks of aerobic exercise can significantly reduce albuminuria in obese diabetic rats, improve renal nitric oxide metabolism, but may induce renal damage under conditions of low nitric oxide bioavailability. Therefore, the protective effects of aerobic training on the kidneys may depend on the bioavailability of nitric oxide (123). Moreover, long-term regular exercise can activate the AMPK/miR-181b axis by increasing blood flow, thereby improving endothelial dysfunction (124). Thus, long-term and sustained exercise is important for improving endothelial cell function and protecting kidney health.

#### 3.2.6 The impact of exercise on dyslipidemia and its progression in patients with DKD

Patients with diabetic kidney disease (DKD) frequently present with dyslipidemia, a pathological condition typically characterized by elevated levels of low-density lipoprotein cholesterol (LDL-C) and triglycerides (TG), alongside reduced levels of high-density lipoprotein cholesterol (HDL-C). Research indicates that these lipid abnormalities play a critical role in the development of DKD. For instance, elevated TG and decreased HDL-C levels are closely associated with the onset and progression of chronic kidney disease (CKD) (125). High TG levels and low HDL-C levels are not only significantly correlated with advanced stages of CKD but may also accelerate the deterioration of DKD through various mechanisms (126).

In the pathological progression of DKD, dyslipidemia plays a key role. Elevated LDL-C and TG levels can damage renal microvascular endothelial cells, leading to increased oxidative stress and inflammatory responses, which in turn promote glomerulosclerosis and tubulointerstitial fibrosis (127). Additionally, lipid deposition in the glomeruli and renal tubules significantly affects kidney structure and function, potentially resulting in proteinuria and declining renal function. Lipid accumulation due to disrupted renal lipid metabolism can induce inflammation and fibrosis, further impairing renal function (128). Lipid deposition in tubular cells may also exacerbate renal injury by affecting cellular energy metabolism, ultimately driving the progression of proteinuria and CKD (129).

Exercise, as an effective non-pharmacological intervention, significantly slows the progression of DKD by improving dyslipidemia. Existing studies have shown that exercise enhances the activity of lipoprotein lipase (LPL), increases hepatic lipid uptake and metabolism, and effectively reduces blood lipid levels. Exercise not only improves systemic lipid metabolism but also reduces renal lipid deposition and associated inflammatory responses, thereby delaying the progression of DKD (130, 131). Specifically, aerobic exercise has demonstrated significant effects in reducing LDL-C and TG levels while increasing HDL-C levels.

A substantial body of research has confirmed the significant efficacy of exercise interventions in improving dyslipidemia in DKD patients. For example, 12 weeks of moderate-intensity aerobic exercise has been shown to significantly lower LDL-C and TG levels in patients with type 2 diabetes while increasing HDL-C levels. In one study, participants engaged in 30 min of aerobic exercise three times a week, resulting in significant reductions in LDL-C and TG levels, and an increase in HDL-C levels after 12 weeks (132). These improvements in lipid parameters not only help reduce cardiovascular disease risk but also slow the progression of diabetes-related complications.

To maximize the improvement of dyslipidemia in DKD patients, individualized exercise intervention programs are particularly important. Studies suggest that moderate aerobic exercise and resistance training, when conducted under medical supervision, can effectively improve lipid levels (133). Personalized exercise programs should be tailored to the patient’s specific conditions, such as age, fitness level, and severity of the disease, to achieve optimal lipid control by lowering LDL-C and TG levels while raising HDL-C levels. Future research should further clarify the optimal strategies for different types and intensities of exercise in lipid management and explore ways to enhance patient adherence to exercise, thereby optimizing lipid management and slowing the progression of DKD.

#### 3.2.7 Physical decline and exercise in diabetic and pre-diabetic CKD

Evidence has shown that DKD is a subtype or specific type of CKD. Thus, it is also necessary to discuss “Physical Decline and Exercise in Diabetic and Pre-Diabetic CKD.” The issue of physical decline in patients with chronic kidney disease is particularly severe among those with diabetes and pre-diabetes. Diabetes complicates the progression of CKD, accelerating renal function decline, especially through the effects of hypertension and metabolic abnormalities (110, 134). Insulin resistance is a key pathological mechanism between diabetes and CKD, leading to increased risk of cardiovascular diseases in patients (135). Furthermore, these patients often experience endothelial dysfunction, such as proteinuria, which is greatly linked to cognitive impairments and physical frailty (110). As chronic kidney disease progresses, with the decline in glomerular filtration rate (eGFR), patients’ physical functions gradually deteriorate. Studies have shown that patients with lower eGFR are more likely to experience declines in cognitive and physical functions (136). Additionally, cardiovascular diseases (CVD) and diabetes also increase the all-cause mortality and physical dysfunction in these patients (135). Accompanying these health issues, the decline in physical capacity in CKD patients not only limits daily activities but also affects their ability to engage in rehabilitation and exercise therapy (135). Despite the common decline in physical function among CKD patients, the role of exercise interventions in delaying this process cannot be overlooked. Existing research indicates that regular aerobic and resistance training can improve glycemic control, insulin sensitivity, and reduce chronic inflammation in CKD patients (137, 138). In prediabetic and diabetic patients, exercise effectively enhances glucose metabolism, boosts muscle strength, and reduces complications associated with cardiovascular diseases (138). Moreover, exercise can also improve cognitive functions in these patients (137). In elderly CKD patients, research has found that regular exercise helps to delay cognitive decline, which is especially important for diabetic and prediabetic patients (72).

### 3.3 Clinical research on the improvement of diabetic kidney disease through exercise

Numerous clinical studies have demonstrated the benefits of exercise in helping diabetic patients control blood sugar, lipids, and weight, improving insulin sensitivity, enhancing cardiovascular and pulmonary function, and reducing the risk of cardiovascular and microvascular complications. This section reviews key studies that have focused specifically on the impact of exercise interventions on diabetic kidney disease (DKD) outcomes (139). Additionally, meta-analyses have shown that exercise has a beneficial effect on improving the renal function of diabetic patients, effectively reducing the incidence of microalbuminuria and renal failure (140). The results of a large randomized controlled trial, T2D Patients with Intensive Glucose Control and Vascular Complications showed that patients who performed moderate to high-intensity exercise had a lower risk of microvascular events compared to those who did not exercise or only performed light physical activity during a median follow-up period of 5 years (141). The FinnDiane study in Finland found that physical activity was related to DKD in T1D patients, and compared to patients with normal urinary albumin excretion rates, those with microalbuminuria had a higher frequency of low-intensity physical activity. After adjusting for confounding factors, higher frequencies and intensities of physical activity were found to reduce the risk of DKD in T1D patients (142). A recent large-sample study in elderly patients with diabetes found that regular physical activity (at least 2 times per week) was significantly associated with lower ESKD and proteinuria rates, as well as a slower decline in GFR (143). In a 39-month prospective follow-up study conducted by Tamiya et al. on a cohort of 173 patients with DKD, it was found that sedentary behavior of ≥525 min/day increases the risk of kidney and cardiovascular events and/or all-cause mortality among DKD patients (144).

The latest studies have shown that a combination of aerobic exercise and quadriceps muscle strengthening training for 6 months can reduce the risk of peritoneal/hemodialysis, cardiovascular disease, and all-cause mortality in DKD patients, while also increasing high-density lipoprotein cholesterol levels and improving lip metabolism (145). These clinical studies have proven the beneficial effects of exercise on DKD, but currently, most studies on exercise intervention in DKD are small clinical trials with short durations, and more large-sample, long-term randomized controlled studies are needed to provide evidence for the exercise guidance of DKD patients.

Despite the abundant evidence showing the benefits of regular exercise for diabetic patients, the majority of diabetic patients have not developed a regular exercise habit. A large cross-sectional study in Europe on 18,028 adult T1D patients showed that less than 20% of patients were able to perform aerobic exercise at least twice a week (146). The results of a self-reported exercise study in American T2D patients showed that only 42.6–65.1% of patients met the American Diabetes Association (ADA) guideline-recommended exercise duration (147).

There is currently a lack of research on the current status of exercise in DKD patients, but epidemiological studies on CKD show that CKD patients average 9 days of exercise per month, and 45% of ESKD patients do not exercise (148). The reasons for the decreased exercise in DKD patients may include difficulty in controlling blood sugar, decreased muscle function, co-morbidities such as cardiovascular disease, and reduced hemoglobin levels due to decreased erythropoietin production. In addition, inadequate exercise counseling by healthcare providers, lack of exercise prescriptions, and incentives are also contributing factors to the lack of exercise in DKD patients (139).

In general, regular exercise has been proven to have numerous benefits for individuals with diabetes, including helping to control blood sugar, lipids, and weight, improving insulin sensitivity, and enhancing cardiovascular and pulmonary function. It also improves overall health and reduces the risk of cardiovascular and microvascular complications and mortality. Exercise has also been shown to have beneficial effects on improving renal function, reducing microalbuminuria, and decreasing the incidence of renal failure. Please refer to Table 1 for clinical research on the improvement of diabetic kidney disease through exercise.

**Table 1:** Clinical research on the improvement of diabetic kidney disease through exercise.

Authors	Publication year	Research object	Exercise intervention		Research findings	Clinical significance for DKD	References
			EG	CG			
ElSayed et al.	2023	Diabetes patients	Not applicable	Not applicable	Blood Glucose: Decreased HbA1c Blood Lipids: Increased HDL, decreased LDL Blood Pressure: Reduced blood pressure Kidney Markers: Increased urinary protein, no DKD worsening DKD Progression: Slowed progression	Diabetes self-management education and support are crucial for improving clinical outcomes, health status, and well-being	(139)
Blomster et al.	2013	Patients with type 2 diabetes	None or mild	Moderate to vigorous	Moderate to vigorous activity associated with reduced risk of cardiovascular events, microvascular complications, and all-cause mortality	Moderate to vigorous activity can reduce the risk of cardiovascular and microvascular complications and mortality in patients with type 2 diabetes	(141)
Pongrac et al.	2022	Type 1 diabetes patients	Not specifically divided into experimental and control groups, but varied levels of LTPA (Leisure Time Physical Activity) were analyzed		Low level of LTPA associated with poor glycemic control. High intensity LTPA does not confer additional benefit on HbA1c level. High frequency and intensity of LTPA reduced risk of CVD events. Intensive physical activity prevents the initiation and progression of diabetic nephropathy. Frequent physical activity reduces the risk of severe diabetic retinopathy.	Emphasizes the importance of avoiding sedentary behavior. Suggests that exercise should be a part of treatment regimen for diabetic kidney disease (DKD). Indicates high-intensity exercise may have mixed effects on diabetic retinopathy.	(142)
Böhm et al.	2022	Diabetic and non-diabetic individuals at high cardiovascular risk	Engaged in moderate exercise	Lower exercise frequency	Moderate exercise inversely associated with renal and cardiovascular risks, effective in both diabetic and non-diabetic patients	Exercise benefits kidney outcomes, effective with at least two sessions per week, particularly crucial for diabetic patients	(143)
Tamiya et al.	2020	Patients with diabetic kidney disease (DKD)	Not specified	Not specified	Extended sedentary time increases the risk of all-cause death and new cardiovascular events	Reducing sedentary time may be an important treatment strategy to prevent cardiovascular events and all-cause death, and may delay the initiation of hemodialysis	(144)
Tamiya et al.	2023	Patients with diabetic kidney disease (DKD)	67 participants received intervention	67 participants did not receive intervention	Long-term tailor-made exercise reduces the risk of cardiovascular diseases and all-cause mortality	Long-term individualized exercise may be an effective means of reducing cardiovascular events and all-cause mortality in DKD patients	(145)
Bohn et al.	2015	Adults with type 1 diabetes	Active group (more than two times per week)	Inactive group (no exercise)	Regular physical activity improves glycemic control and cardiovascular risk factors without increasing the risk of adverse events	Regular physical activity may be effective in glycemic control and cardiovascular risk management in patients with type 1 diabetes	(146)
Bazargan-Hejazi et al.	2017	U.S. adults with type 2 diabetes mellitus	No detailed information	No detailed information	Racial/ethnic disparities in self-reported vs. objectively measured physical activity; African Americans had the lowest compliance in both measurements; Whites overestimated, Hispanics underestimated their activity levels	Enhances understanding of racial/ethnic differences in physical activity perception and reporting, which may aid in diabetes management and prevention of DKD	(147)
Mallamaci et al.	2020	Patients with chronic kidney disease (CKD)	Individualized, low-intensity home-based exercise program	Standard care	Exercise training improved eGFR, BMI, blood pressure control, and quality of life in CKD patients	Exercise training may benefit health status and quality of life in CKD patients, potentially aiding in the management and prevention of diabetic kidney disease (DKD)	(148)

This table compiles recent clinical studies on the effects of exercise interventions in improving diabetic kidney disease (DKD). The table is categorized by study authors, publication year, research subjects, exercise interventions, research findings, and the clinical significance for DKD. The studies presented in the table provide clinicians and researchers with detailed information on the impact of exercise on patients with DKD, aiding in clinical decision-making and guiding future research directions. EG, Experimental Group; CG, Control Group; HbA1c, Glycated Hemoglobin; HDL, High-Density Lipoprotein; LDL, Low-Density Lipoprotein; CVD, Cardiovascular Disease; LTPA, Leisure Time Physical Activity; eGFR, Estimated; Glomerular Filtration Rate; BMI, Body Mass Index.

### 3.4 Potential exercise prescriptions for DKD

According to the American Diabetes Association (ADA)’s Lifestyle and Healthcare Guidelines released in 2023, patients with diabetes should engage in diversified exercises including aerobic exercise, resistance exercise, combination exercise, and flexibility exercise (139). The specific recommendations are as follows: children and adolescent diabetes patients should engage in 60 min of moderate to vigorous intensity aerobic exercise daily, and at least 3 sessions of high-intensity resistance exercise per week; adults should engage in at least 150 min of moderate to vigorous intensity aerobic exercise per week and perform resistance exercise 2–3 times per week; elderly individuals should participate in 2–3 sessions of flexibility training and balance training per week (139). Resistance exercise and high-intensity interval training (HIIT) are not recommended for elderly patients (149). It is worth noting that ADA’s guidelines on physical activity/exercise do not restrict the exercise modalities for diabetes patients (150) but recommend individuals with microalbuminuria to participate in moderate to vigorous intensity exercise. For individuals with macroalbuminuria, exercise should start with low-intensity, low-impact activities and gradually increase intensity and duration. In 2022, the Kidney Association released clinical practice guidelines for exercise and lifestyle management in patients with chronic kidney disease (CKD), providing relevant recommendations for non-dialysis CKD patients, dialysis patients, and kidney transplant patients, encouraging them to break sedentary behavior and gradually increase exercise intensity, performing 150 min of moderate-intensity aerobic exercise per week or 75 min of moderate to vigorous intensity exercise (149).

A large body of research has confirmed the efficacy and safety of exercise in DKD patients, and thus, the guidelines recommend that dialysis patients with no contraindications engage in 150 min of moderate-intensity exercise or 75 min of high-intensity exercise per week (150). Early studies have demonstrated the benefits of different forms of exercise in diabetic kidney disease (DKD), with aerobic training being the most effective for enhancing cardiorespiratory fitness, improving insulin sensitivity, reducing weight, and improving glycemic and lipid metabolism; resistance exercise and HIIT can increase skeletal muscle mass and decrease the risk of exercise-induced hypoglycemia in individuals with type 1 diabetes (T1D) (151). Interestingly, comparing the effects of different exercise modalities, including aerobic, resistance, combination, and HIIT, on DKD, it was found that they can improve microvascular vasodilation in patients with type 2 diabetes (T2D), with aerobic and combination exercise having the most significant improvement on endothelial function (118); HIIT and moderate-intensity continuous aerobic exercise can significantly enhance activation of the renin-angiotensin-aldosterone system (RAAS) regulatory axis, with the latter having a more pronounced effect (152). However, the optimal exercise modality and regimen for DKD have not been established globally, and healthcare professionals should actively encourage patients to participate in exercise, select appropriate exercise modalities based on individual conditions, and gradually increase exercise intensity.

When prescribing exercise for patients with diabetic kidney disease (DKD), healthcare professionals need to carefully consider the patient’s overall health status and any potential complications to tailor appropriate exercise recommendations. In certain cases, exercise may not be suitable for all patients, particularly for those with proliferative diabetic retinopathy or severe non-proliferative diabetic retinopathy (88), cardiovascular disease including myocardial ischemia, heart failure, or peripheral arterial disease, patients with autonomic neuropathy resulting in impaired regulation of heart rate and blood pressure (153), as well as those with peripheral neuropathy and foot ulcers. In such cases, the type and intensity of exercise should be carefully considered, and it may be necessary to avoid or reduce exercise intensity.

Additionally, patients with type 1 diabetes (T1D) are at a higher risk of experiencing fluctuations in blood glucose levels during and after exercise, which increases the risk of hypoglycemia. To minimize this risk, patients should select appropriate types of exercise, control the intensity and duration of exercise, adjust insulin doses, consume additional carbohydrates, and continuously monitor blood glucose levels before, during, and after exercise to enhance glycemic control (154).

Exercise recommendations for DKD patients also vary depending on their age and health status. For children and adolescents with diabetes, it is recommended to engage in 60 min of moderate to vigorous aerobic exercise daily, with at least three sessions of high-intensity resistance exercise per week. Adults are advised to engage in at least 150 min of moderate to vigorous aerobic exercise per week, along with 2–3 sessions of resistance exercise. For older adults, 2–3 sessions of flexibility and balance training per week are recommended. Dialysis patients are advised to engage in 150 min of moderate-intensity exercise or 75 min of vigorous-intensity exercise per week. Healthcare professionals should select suitable exercise modalities based on the patient’s specific health status and physical condition and gradually increase exercise intensity to ensure safety and effectiveness. In conclusion, exercise recommendations for DKD patients should be individualized to meet the needs of different patients while minimizing the risk of complications and other potential hazards. These potential exercise prescription recommendations for patients with DKD are represented in Table 2.

**Table 2:** Potential exercise prescription recommendations for people with DKD.

Patient category	Exercise type and intensity	Frequency and duration	Special considerations
Children and adolescents (139, 150)	Moderate to vigorous intensity aerobic exercise	60 min daily	–
	High-intensity resistance exercise	At least 3 sessions per week	–
Adults (139, 150)	Moderate to vigorous intensity aerobic exercise	At least 150 min per week	–
	Resistance exercise	2–3 times per week	–
Elderly individuals (139, 149)	Flexibility training and balance training	2–3 sessions per week	Avoid resistance exercise and high-intensity interval training (HIIT)
Dialysis patients (150)	Moderate-intensity exercise or high-intensity exercise	150 min of moderate or 75 min of high intensity per week	No contraindications
Type 1 diabetes (T1D) patients (151, 154)	Exercise modality based on individual condition	Adjust intensity, duration, and insulin doses	Monitor blood glucose levels to prevent hypoglycemia
Patients with microalbuminuria (150)	Moderate to vigorous intensity exercise	Adjust according to condition	–
Patients with macroalbuminuria (150)	Start with low-intensity, low-impact activities	Gradually increase intensity and duration	–
Patients with contraindications (88, 153)	Avoid or reduce exercise intensity	Based on individual health status	Includes retinopathy, cardiovascular diseases, autonomic neuropathy, peripheral neuropathy, and foot ulcers

This table summarizes exercise prescription recommendations for various types of patients with diabetic kidney disease (DKD), including children and adolescents, adults, the elderly, dialysis patients, patients with type 1 diabetes, those with microalbuminuria, macroalbuminuria, and those with contraindications. The content is organized by patient category, providing the recommended type and intensity of exercise, frequency and duration, as well as any special considerations. These guidelines are designed to assist clinicians in creating personalized exercise plans for DKD patients with different health conditions, with the goal of promoting overall health and preserving kidney function.

## 4 Conclusion

### 4.1 Summary of current research progress

In conclusion, exercise has shown promising potential as a therapeutic approach to alleviate DKD. Exercise can improve blood glucose stability associated with DKD, RAAS, reduce renal oxidative stress and inflammation, enhance the crosstalk between muscle and kidney, and improve endothelial cell function. These mechanisms contribute to the overall improvement of DKD. Exercise offers several advantages over traditional treatment methods, including its safety, effectiveness, and lack of apparent side effects. It can be used as an adjunct treatment to medication, blood glucose control, protein-restricted diet, and blood pressure management. Despite the clear benefits of exercise in DKD management, there is still a lack of large-scale, long-term randomized controlled trials to provide more evidence and establish exercise guidelines specific to DKD. Healthcare professionals should actively encourage DKD patients to engage in exercise and prescribe personalized exercise regimens based on individual conditions.

### 4.2 Prospects for future research

Future research should focus on exploring optimal exercise modalities, intensities, and durations for DKD patients, as well as investigating the long-term effects of exercise on DKD outcomes. Additionally, efforts should be made to address the barriers and challenges that prevent DKD patients from engaging in regular exercise, such as lack of awareness, motivation, and guidance. Therefore, exercise holds great potential as a non-pharmacological intervention for DKD and can significantly improve the quality of life for DKD patients. By integrating exercise into the management of DKD, we can reduce the burden on families and societies and ultimately improve the overall clinical outcomes of DKD patients.
